# The Context Assessment for Community Health tool - investigating why what works where in low- and middle-income settings

**DOI:** 10.1186/1472-6963-14-S2-P8

**Published:** 2014-07-07

**Authors:** Anna Bergström, Hoa Dinh, Duc Duong, Jesmin Pervin, Anisur Rahman, Sarah Skeen, Mark Tomlinson, Peter Waiswa, Elmer Zelaya, Lars Wallin

**Affiliations:** 1 International Maternal and Child Health, Department of Women’s and Children’s Health, Uppsala University, Uppsala, Sweden; 2 Research Institute for Child Health, National Hospital of Paediatrics, Hanoi, Viet Nam; 3 Hanoi School of Public Health, Hanoi, Viet Nam; 4 Centre for Reproductive Health, International Centre for Diarrhoeal Disease Research, Dhaka, Bangladesh; 5 Department of Psychology, Stellenbosch University, Stellenbosch, South Africa; 6 School of Public Health, Makerere University College of Health Sciences, Kampala, Uganda; 7 Fundacion Coordinación de Hermanamientos e Iniciativas de Cooperación (CHICA), León, Nicaragua; 8 School of Health and Social Studies, Dalarna University, Falun, Sweden; 9 Department of Neurobiology, Care Sciences and Society, Division of Nursing, Karolinska Institutet, Stockholm, Sweden

## Background

The gap between what is known and what is practiced results in patients not benefitting from advances in healthcare and unnecessary costs for clients and health systems. The Promoting Action on Research Implementation in Health Services (PARIHS) framework posits (1) strong evidence, (2) context in terms of coping with change, and (3) facilitation as elements influencing successful implementation of new knowledge [1]. A strong context is considered key to warrant an environment receptive to change. Tools for systematic mapping of aspects of context influencing implementation have been developed for, and are being used in, high-income settings whereas there are no tools available for this purpose for low- and middle-income countries (LMICs).

## Materials and methods

The development of the Context Assessment for Community Health (COACH) tool departed from the PARIHS framework and was undertaken in Bangladesh, Vietnam, Uganda, South Africa and Nicaragua in six phases; (1) defining dimensions and draft tool development, (2) quantitative and qualitative content validity amongst in-country experts, (3) content validity amongst international experts, (4) response process, (5) translation and (6) evaluation of psychometric properties. The tool has been validated for use amongst physicians, nurse/midwives and community health workers in these five settings.

## Results

This study indicates that dimensions of context identified to influence implementation in high-income healthcare settings are also relevant in LMICs. Having said this, there are additional aspects of context of relevance in LMICs. The final version of the tool includes 49 items measuring the following eight aspects of context: leadership, work culture, monitoring services for action, sources of information, resources, community engagement, commitment to work and informal payment.

## Conclusions

Application of the COACH tool will allow for systematic characterization of local healthcare context prior to or as part of the evaluation of implementing new interventions and allow for deepened insights into the black-box of implementation in LMICs.
